# iPSCs derived from infertile men carrying complex genetic abnormalities can generate primordial germ-like cells

**DOI:** 10.1038/s41598-022-17337-2

**Published:** 2022-08-22

**Authors:** Aurélie Mouka, Brahim Arkoun, Pauline Moison, Loïc Drévillon, Rafika Jarray, Sophie Brisset, Anne Mayeur, Jérôme Bouligand, Anne Boland-Auge, Jean-François Deleuze, Frank Yates, Thomas Lemonnier, Patrick Callier, Yannis Duffourd, Patrick Nitschke, Emmanuelle Ollivier, Arnaud Bourdin, John De Vos, Gabriel Livera, Gérard Tachdjian, Leïla Maouche-Chrétien, Lucie Tosca

**Affiliations:** 1 AP-HP, Université Paris-Saclay-Hôpital Antoine Béclère, Service d’Histologie, Embryologie et Cytogénétique, 92140 Clamart, France; 2 Faculté de Médecine, Université Paris-Saclay, 94270 Le Kremlin-Bicêtre, France; 3 Inserm U1287, Laboratoire Cellules Souches Hématopoïétiques et Hémopathies Myeloïdes, Université Paris-Saclay, Gustave Roussy Cancer Campus, 94800 Villejuif, France; 4 Laboratoire de Développement des Gonades, UMRE008 Stabilité Génétique Cellules Souches et Radiations, Commissariat à l’Energie Atomique et Aux Énergies Alternatives, Institut de Biologie François Jacob, 92265 Fontenay-aux-Roses, France; 5 Université de Paris, Paris, France; 6 Université Paris-Saclay, 91400 Orsay, France; 7 AP-HP Sorbonne Université-La Pitié Salpêtrière, SiRIC Curamus, 75013 Paris, France; 8 Sup’Biotech/ Laboratoire CEA-IBFJ-SEPIA, 92265 Fontenay-aux-Roses, France; 9 INSERM UMR_S U1185, Faculté de Médecine Paris-Saclay, Université Paris-Saclay, Le Kremlin Bicêtre, France; 10 Service de Génétique Moléculaire, Pharmacogénétique et Hormonologie, Hôpitaux Universitaires Paris Sud, AH-HP, CHU Bicêtre, Paris, France; 11 Centre National de Recherche en Génomique Humaine, Université Paris-Saclay, CEA, 91057 Evry, France; 12 Département de Génétique Humaine, Hôpital Universitaire de Dijon, Dijon, France; 13 Inserm UMR 1231 GAD, Faculté des Sciences de la Santé, Université de Bourgogne et de Franche-Comté, Dijon, France; 14 Plateforme Bio-Informatique, IMAGINE Institute, Université Paris Descartes, Paris, France; 15 PhyMedExp, Université Montpellier, INSERM, CHU Montpellier, Montpellier, France; 16 IRMB, Université Montpellier, INSERM, CHU Montpellier, Montpellier, France; 17 Laboratoire des Mécanismes Moléculaires et Cellulaires des Maladies Hématologiques et leurs Implications Thérapeutiques; INSERM U 1163, Institut IMAGINE, Paris, France; 18 Division des Thérapies Innovantes, CEA, Institut de Biologie François Jacob, 92260 Fontenay-aux-Roses, France; 19 AP-HP, Université Paris-Saclay - Hôpital Antoine Béclère, Biologie de la Reproduction, 92140 Clamart, France

**Keywords:** Developmental biology, Genetics, Stem cells, Diseases, Medical research

## Abstract

Despite increasing insight into the genetics of infertility, the developmental disease processes remain unclear due to the lack of adequate experimental models. The advent of induced pluripotent stem cell (iPSC) technology has provided a unique tool for in vitro disease modeling enabling major advances in our understanding of developmental disease processes. We report the full characterization of complex genetic abnormalities in two infertile patients with either azoospermia or XX male syndrome and we identify genes of potential interest implicated in their infertility. Using the erythroblasts of both patients, we generated primed iPSCs and converted them into a naive-like pluripotent state. Naive-iPSCs were then differentiated into primordial germ-like cells (PGC-LCs). The expression of early PGC marker genes *SOX17*, *CD-38*, *NANOS3*, *c-KIT*, *TFAP2C*, and *D2-40*, confirmed progression towards the early germline stage. Our results demonstrate that iPSCs from two infertile patients with significant genetic abnormalities are capable of efficient production of PGCs. Such in vitro model of infertility will certainly help identifying causative factors leading to early germ cells development failure and provide a valuable tool to explore novel therapeutic strategies.

## Introduction

Infertility is a major public health issue, affecting 10–15% of couples in reproductive age worldwide. In half of the cases, infertility is due to a male factor^1,2^ with varying degrees of severity. The most extreme form is non-obstructive azoospermia (NOA), which is characterized by the complete absence of sperm in the ejaculate. Genetic causes have been attributed to microdeletions in the azoospermia factor (AZF) region^3^, chromosomal abnormalities, single gene mutations, or multifactorial inheritance^4^. Disorders of sex development (DSD) are rare congenital conditions characterized by a complete or partial mismatch between genetic and phenotypic sex, and represent another cause of infertility^5^. One of these DSD is the XX male syndrome, a rare congenital intersex condition in individuals with male phenotype expression and variable clinical presentations, ranging from ambiguous to normal male genitalia and infertility^6,7^. The fate of the testes is determined by the sex-determining region Y (*SRY*) gene in the Y chromosome. Approximately 80% of 46,XX DSD patients are *SRY*-positive, with most cases resulting from *SRY* gene translocation onto the pseudo-autosomal region PAR1 of one X chromosome^8^. In contrast, the genetic causes of most cases of 46,XX *SRY*-negative DSD patients remain unknown.

Research on gametogenesis responds to the actual need for reproductive health. Development of biological models to comprehensively reproduce the early stages of germ cell differentiation would enable a better understanding of the molecular mechanisms regulating normal spermatogenesis and those underlying infertility. In mammals, primordial germ cells (PGCs) are the precursors of germline lineage that give rise to gametes^9,10^. In mice, signals from the proximal extra-embryonic ectoderm (BMP4 and BMP8b) and the visceral endoderm (BMP2) play an essential role in inducting PGCs^10^. In particular, BMP4, which is produced by extra-embryonic mesoderm, controls the survival and localization of PGCs^11^. PGC specification is characterized by multiple key events; conversion of 5mC into 5-hydroxymethylcytosine (5hmC) by Ten-eleven translocation proteins would constitute the first step of the DNA demethylation pathway^12^. However, little is known about PGC formation in humans due to poor accessibility.

In the stem cell research field, induced pluripotent stem cells (iPSCs) provide an opportunity to reproduce in vitro pathological processes and open new avenues to understand the pathological states at a molecular level. Many groups have attempted to study normal human germ cell differentiation using human embryonic stem cells (hESCs) or hiPSCs with varying degrees of success, eventually resulting in breakthroughs in the field^13–21^. However, the generation of PGCs in the context of infertility has been poorly addressed. The most impressive work reported so far was performed on iPSCs derived from infertile male mice with trisomy XXY or XYY. In the aforementioned study, trisomic cells were demonstrated to lose their extra chromosome through the reprogramming process, with generated sperm giving rise to healthy and fertile offspring^22^. To date, very few studies have explored hiPSCs in human male infertility and only one recent study reported the generation of germ cell-like cells from azoospermic men carrying microdeletions in the AZF region^23^. Generating patient-specific iPSCs with the same genetic background as infertile donors provide unprecedented human models for studying genetic disorders of infertility; and assessing their potential to differentiate into PGC-LCs is an essential step in understanding infertility.

The purpose of this study was to determine whether infertile men could produce PGCs within the framework of genetic disorders. Genetic abnormalities of two patients were analyzed using conventional cytogenetic and molecular techniques as well as whole genome sequencing. Several genes potentially implicated in the infertility and/or DSD of the patients were identified. Subsequently, patient erythroblasts were reprogrammed into primed iPSCs to obtain the unique cellular model that enables assessment of the ability of each patient's genetic program to produce PGCs. Primed iPSCs were first converted into a naive-like state before inducing their differentiation into germinal cells. Our results show that the iPSCs of both patients give rise to PGC-like cells (PGC-LCs), indicating that infertile men with distinct genetic abnormalities are capable of robust production of PGCs. This study also demonstrates that iPSC technology provides a powerful biological tool to identify early events of embryonic development that may be responsible for infertility and could be used to explore innovative therapeutic strategies. Further specific investigations are needed to determine whether these PGC-LCs can form functional and fertile gametes, but this remains a challenging task, especially for human PGCs, due to the many ethical considerations that are raised in this field.

## Results

### Conventional cytogenetic and molecular analysis

**Figure 1:**
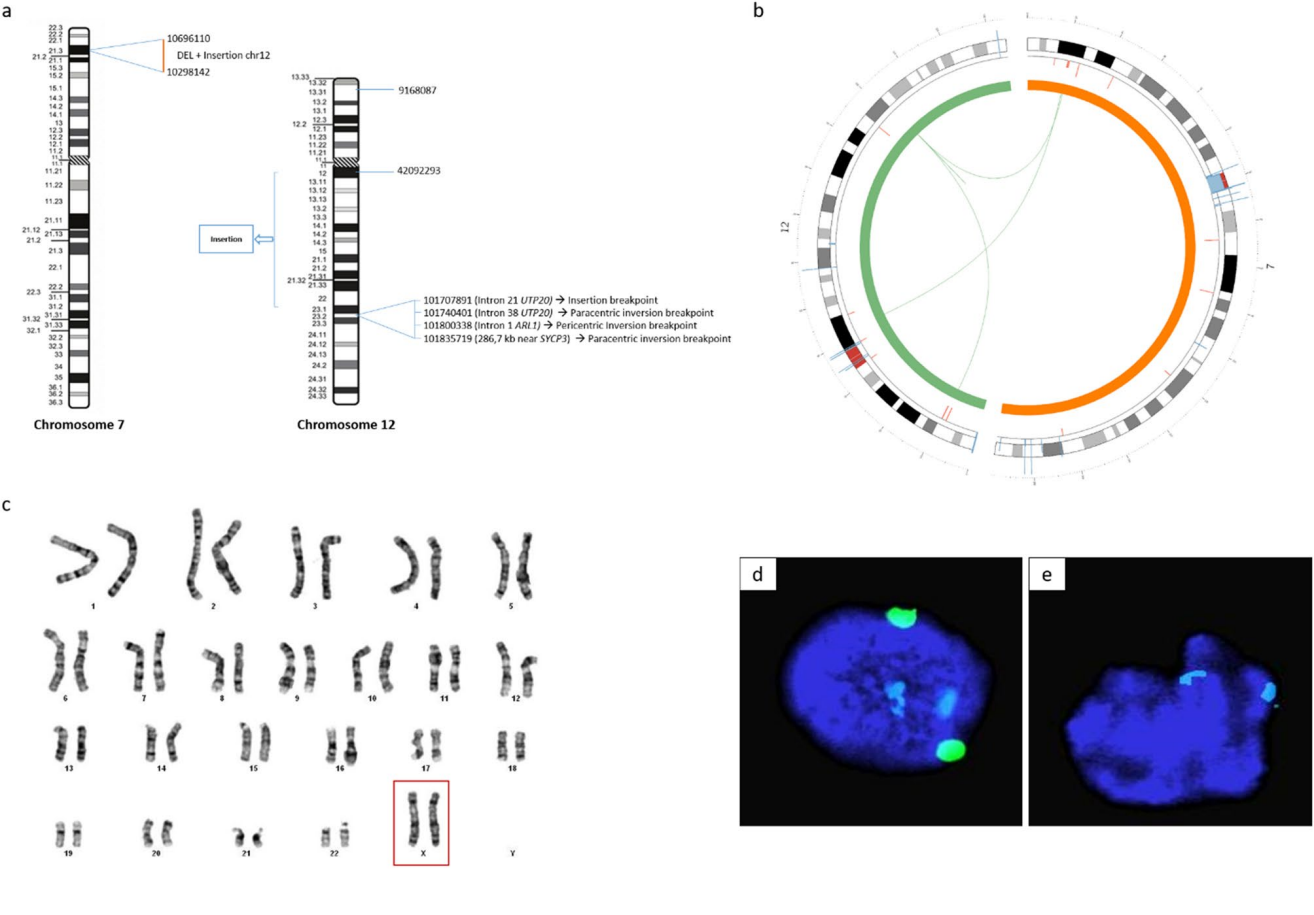
Short-read Whole genome sequencing results of patient 1 and conventional and molecular cytogenetic analysis for patient 2. (**a**) The CCR of patient 1 involved 7 breakpoints: one on the short arm of the derivative chromosome 7 7p21.3 region along with two deletions (chr7:11,801,000–11,791,000 and chr7:10,257,000–10,656,000), one on the short arm of the derivative chromosome 12 12p13.31 region (chr12:9,015,000–9,015,001) and the remaining 5 breakpoints on the long arm of the derivative chromosome 12 (chr12:41,698,000–41,698,001 proximal 12q12 breakpoint; chr12:101,314,000–101,314,001, chr12:101,346,000–101,346,001, chr12:101,406,000–101,406,001, chr12:101,441,000–101,441,001 distal 12q23.2 breakpoints). Genomic positions are described according to the hg38 genome assembly. (**b**) Circos plot showing the 3 events of the CCR of patient 1: the insertion and the pericentric and paracentric inversions. (**c**) G-banded standard karyotype designated as 46,XX (patient 2). (**d**) FISH analysis on buccal swab using centromeric probe for chromosome 18 (blue, 2 signals), chromosomes X (green, 2 signals) and chromosome Y (red, no signal) (patient 2). (**e**) FISH analysis on buccal swab using centromeric probe for chromosome X (blue, 2 signals), chromosome Y heterochromatic region (green, no signal) and SRY gene locus on chromosome Y (red, no signal) (patient 2).

Patient 1 had non-obstructive azoospermia (see Methods for the complete clinical description) and carried a complex chromosomal rearrangement (CCR) involving chromosomes 7 and 12^24^. Additional results using whole genome sequencing enabled the identification of a total of seven breakpoints and one deletion located at the site of the 7p21.3 breakpoint (Fig. 1a). These breakpoints were associated with three events: insertion of a segment of the long arm of chromosome 12 (12q12q23.2) into the short arm of chromosome 7 (7p21.3), a pericentric inversion of chromosome 12 involving the 12p13.31q23.2 segment, and a paracentric inversion of chromosome 12 involving the 12q23.2 segment (Fig. 1a,b). The deletion identified within the 7p21.3 breakpoint does not involve genes of interest. In the 12q23.2 region, four breakpoints were identified, with two occurring within the *UTP20* and *ARL1* genes; however, these two genes are not related to reproductive function (Fig. 1a). Chromosome 12 breakpoint mapping enabled the identification of a candidate gene, *SYCP3*, encoding an element of the synaptonemal complex that is essential for chromosomal pairing during Prophase I of meiosis, located approximatively 300 kb from the chr12:101,835,719 breakpoint (Fig. 1a). In addition, 1 M array-CGH, as well as a custom-designed CGH-array were performed; however, significant DNA copy number variations that may account for non-obstructive azoospermia were not identified.

Patient 2 is a male with DSD, including ambiguous genitalia (see Methods for the complete clinical description). Conventional analysis revealed a karyotype 46,XX (Fig. 1c). Fluorescence in situ hybridization (FISH) performed on lymphocytes and buccal swab using probes of chromosome 18, X and Y confirmed the absence of chromosome Y material (Fig. 1d). FISH and PCR analysis confirmed the absence of *SRY* gene in patient’s genome (Fig. 1e and data not shown, respectively). The 1 M array-CGH and the custom-designed array-CGH, also failed to identify *SRY* sequences, as well as significant DNA copy number variation that may account for the DSD phenotype.

Next, whole genome sequencing (WGS) analysis was performed for patient 2, revealing three rare variants of potential interest, which comprised: three single-nucleotide polymorphisms (SNPs) (Table 1). The first SNP identified is a “probably damaging” heterozygous missense variant (Polyphen) in exon 3 of the *AMH* gene (c.482G>A) and is associated with persistent Müllerian duct syndrome. This disorder affects normally virilized males who develop female sex organs (uterus and fallopian tubes) due to persistence of Mullerian duct derivatives during foetus development (#OMIM261550). This syndrome is caused by heterozygous mutation inactivating the genes encoding for AMH or AMH receptor (AMHR2) (#OMIM261550). The second SNP is a heterozygous missense mutation (c.1030G>A) in exon 12 of the *NUP107* gene. Mutations in this gene have been reported to cause XX gonadal dysgenesis (#OMIM618078). We also identified a “possibly damaging” heterozygous missense mutation (c.691G >A) in *STAT5B*, which is known to act in many biological processes, including growth hormone insensitivity (#OMIM618985). Thus, WGS highlights rare genetic variants that could assist in deciphering the complex phenotypic traits of the DSD patient.

**Table 1:** Candidate variants from patient 2 whole genome sequencing.

Gene	NUP107	AMH	STAT5B
Chromosome	12	19	17
Position (hg19)	69,109,467 (exon 12)	2,250,720 (exon 3)	40,371,472 (exon 7)
Type	SNP	SNP	SNP
Nucleotide change	c.1030G>A	c.482G>A	c.691G>A
Name	rs778710781	rs778532660	rs917567542
DNA variant	Missense	Missense	Missense
Inheritans, zygosity	AR, He	AR, He	/, He
Associated Phenotype (MIM number)	Ovarian Dysgenesis (618,078)	Persistent Müllerian duct syndrome, type I (261,550)	Growth hormone insensitivity with immunodeficiency (245,590)
**Pathogenecity prediction**			
Polyphen	Benign	Probably damaging	Possibly damaging
SIFT	Tolerated	Tolerated	Tolerated
**Frequency**			
DB %	0.002	0.002	0.004
gnomAD allele %	0.00002122	0.00001494	0.00003494
**Molecular confirmation**			
Sanger	Detected heterozygous	-	Detected heterozygous
Targeted sequencing	-	Detected heterozygous	-

*AD* autosomal dominant, *AMH* anti-Müllerian hormone, *AR* autosomal recessive, *Del* deletion, *He* heterozygous, *Ho* homozygous, *NUP107* nucleoporin 107, *SNP* single nucleotide polymorphism, *STAT5B* signal transducer and activator of transcription 5B.

### Primed iPSCs generation and characterization

**Figure 2:**
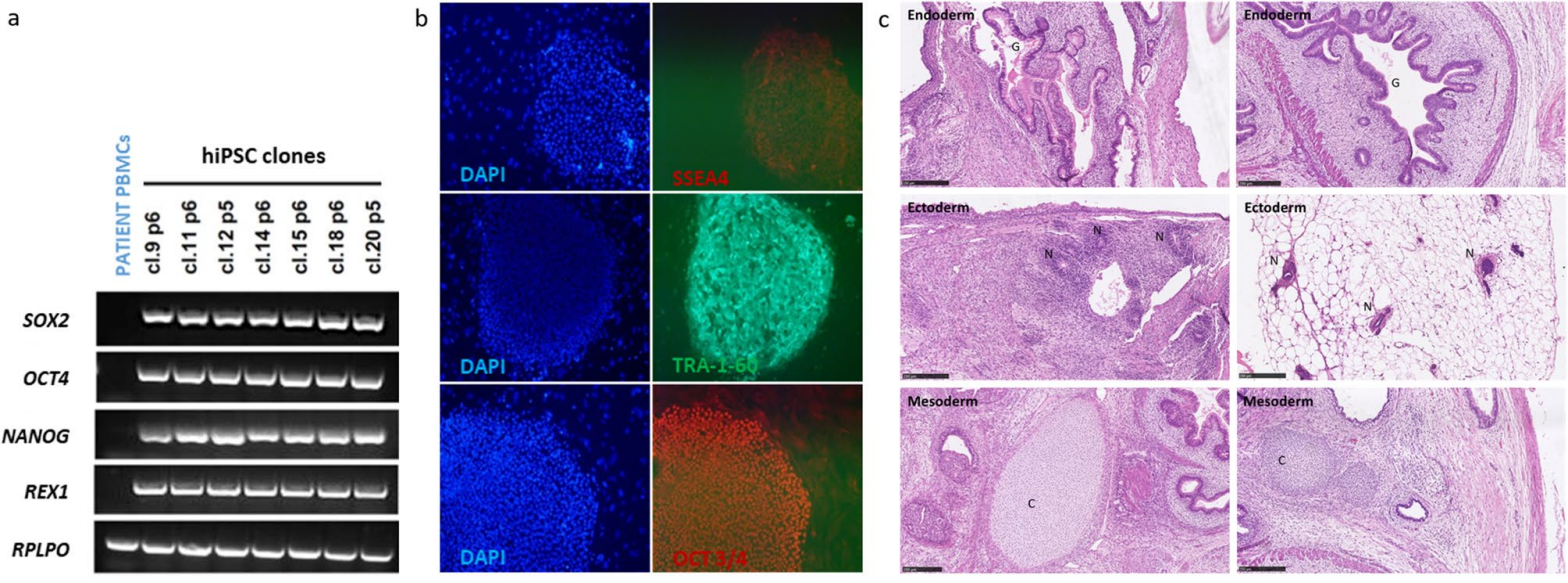
Generation and characterization of hiPSCs. (**a** and **b**) Endogenous expression of pluripotency-related markers by patient 2-derived iPSCs: (**a**) RT-PCR analysis for detection of the pluripotency markers *SOX2*, *OCT4*, *NANOG* and *REX-1*. Full length gels are showed in Supplementary Fig. S5; (**b**) Immunofluorescence staining of three stem cell proteins (SSEA4, TRA-1–60 and OCT3/4) in patient iPS clones 15 and 20. (**c**) Germ cell layer components within teratomas obtained with patient 2-derived iPSCs. The differentiation, at passage 8 (cl.15) and 7 (cl.20), of iPSCs into ectoderm, endoderm and mesoderm was evaluated on whole sections stained with hematoxylin and eosin, for iPS clones 15 (left panel) and 20 (right panel). Hematoxylin and eosin stain, X20. Images of immunofluorescence staining were analyzed using MetaMorph (Molecular Devices, https://fr.moleculardevices.com) and ImageJ 1.52 (National Institutes of Health, https://imagej.nih.gov) Softwares. *C* cartilage, *EB* embryoid body, *G* gut epithelial tissue, *hiPSC* human induced pluripotent stem cells, *N* neural tissue.

Primed iPSCs from DSD patient (patient 2) were generated from erythroblasts following the protocol previously described for patient 1^24^. We investigated the pluripotency of five patient 2 primed iPSCs clones, by analyzing the expression of the endogenous pluripotency marker genes *SOX2*, *OCT4*, *NANOG* by RT-PCR. The pluripotency markers were strongly expressed in all five iPSC clones but not in the primary cells (PBMCs) of the patient (Fig. 2a). Immunofluorescence revealed specific staining for SSEA4, TRA-1–60, and OCT3/4 pluripotency markers in iPSC colonies (Fig. 2b). Therefore, the iPSC clones derived from the infertile DSD patient expressed conventional pluripotency markers, indicating successfully reprogramming.

To ascertain pluripotency, we explored the teratoma-forming potential for two iPSC clones: patient 2 cl.15 and cl.20. Patient 1 cl.12 and cl.32 were described previously^24^. Histological analysis of the tumors produced revealed differentiation of the cells into endodermal, ectodermal and mesodermal tissues, with a histologic mix of glandular gut-like epithelium, neural tissue and large cartilaginous areas (Fig. 2c). The tissues were well-differentiated in all the structures observed and without malignancy.

As a consequence, both in vitro testing for pluripotency markers and in vivo testing for teratoma formation confirmed the pluripotent state and efficient reprogramming of the iPSC clones analyzed.

To assess iPSCs genomic stability, karyotypes of both iPSCs lines were performed. Results showed that the complex chromosomal rearrangement involving chromosome 7 and 12 of patient 1 was still present as well as the 46,XX karyotype of patient 2 (Supplementary Fig. S1). And mainly, it demonstrates that iPSCs reprogramming process did not induce additional chromosomal abnormalities. In order to investigate the possible CNV instability during somatic reprogramming, we performed pangenomic 1 M array-CGH. CNV analysis did not identify significant change of copy number variation.

### Conversion of primed hiPSCs into a naive state of pluripotency

**Figure 3:**
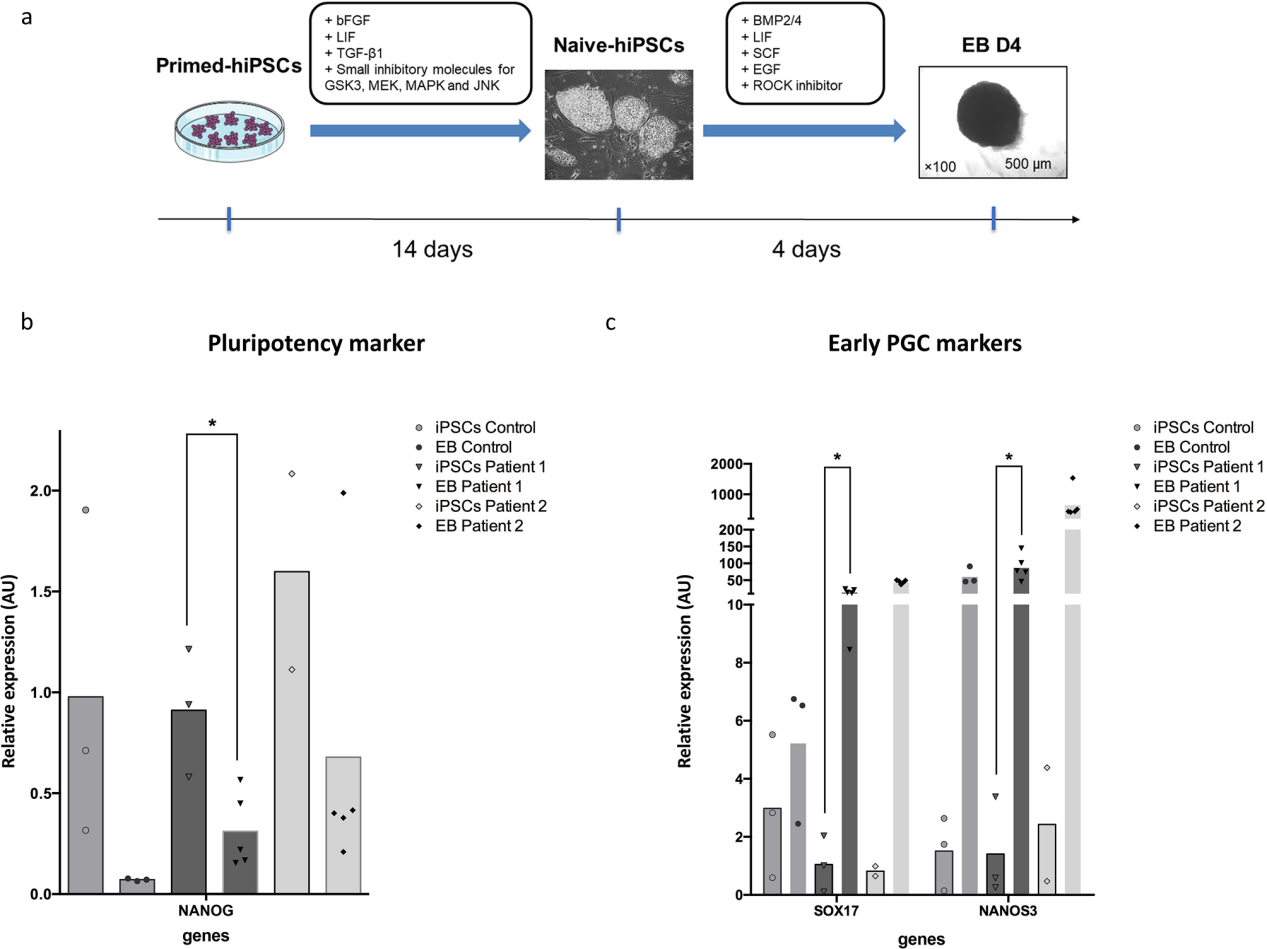
Induction of naive iPSCs state, EB differentiation and gene expression analysis. Timeline of in vitro conversion of primed-hiPSCs into naive-hiPSCs and PGC-LCs differentiation (**a**). RT-qPCR analysis for detection of the pluripotency marker *NANOG* (**b**); early PGC marker genes *SOX17* and *NANOS3 *(**c**). Relative expression levels are shown with normalization to *RPLPO* gene. Histograms represent mean from independent biological replicates represented by each point. Mann–Whitney tests were used for PCR analysis. Mann–Whitney tests were performed to compare expression values. *p < 0.05. *BMP2/4* bone morphogenetic protein 2/4, *EB* embryoid body, *EB D4* embryoid body day 4, *EGF* epidermal growth factor, *GSK3* glycogen synthase kinase-3, *hiPSCs* human induced pluripotent stem cells, *iPSCs* induced pluripotent stem cells, *JNK* c-Jun N-terminal kinase, *LIF* leukemia inhibitory factor, *MAPK* mitogen-activated protein kinases, *MEK* mitogen-activated protein kinase kinase, *SCF* stem cell factor, *TGF-β1* transforming growth factor β1.

Pluripotent stem cells exist in a naive or primed state, respectively represented by pre-implantation epiblast stem cells and developmentally more advanced post-implantation epiblast stem cells (EpiSCs). Primed state represents a more advanced “differentiated” state of pluripotency than the naive state and show lower differentiation efficiency^21,25,26^. For this reason, we focused on converting primed hiPSCs into a naive state as described in Fig. 3a. The protocol for naive iPSC conversion was performed as reported by Gafni et al., 2013^27^. Three clones of patient 1 hiPSCs and two clones of patient 2 hiPSCs were subjected to conversion by providing exogenous stimulation with bFGF, LIF, TGFβ, and the small molecule inhibitor of GSK3, MEK, MAPK, and JNK, termed 4i medium, for the induction and maintenance of human naive stem cell pluripotency. After 2 weeks of culture and maintenance through single-cell dissociation passages, naive state conversion generated cells exhibiting naive-like iPSC characteristics with the morphology changing from flat to domed. This specific phenotype of the colonies and their ability to tolerate single-cell dissociation is a strong indication of successful conversion of primed iPSCs to a naive state but we also assessed the expression of *KLF4* and *TFCP2L1* genes known to be expressed in the naive state^28^. RT-PCR results showed a weak increase of *KLF4* which is already highly expressed in primed iPSCs, but a significant increase of *TFCP2L1* expression in patient naive iPSCs compared to the primed cells, confirming the successful conversion of the patient’s iPSCs from primed to naive state (Supplementary Fig. S2).

### Embryoid body formation and PGC-LCs differentiation

To assess the germline differentiation potential of the patient’s naive hiPSCs, embryoid body (EB) aggregation in 96-well low-attachment plates was performed in the presence of BMP2/4, LIF, SCF, and EGF to induce hPGC-LC differentiation (Fig. 3a). BMP2/4 have been described to promote PGC differentiation from PSCs in EB cultures^10,11^. When transferred into PGC medium, cells within EBs differentiated into PGC-LCs within 4 days. Next, we assessed the presence of germ cells in the EBs by RT-qPCR and immunohistochemistry.

RT-qPCR were performed to assess the expression of *NANOG* pluripotency gene, *NANOS3* and *SOX17* early PGC markers on undifferentiated naive iPSCs and EBs at day-4 of differentiation from a control, patient 1 and patient 2. The results showed statistically significant decrease of *NANOG* in the EBs of patient 1, as well as the increased expression of the early PGC markers *NANOS3* and *SOX17*, the critical specifier of human PGC-LCs as compared to the naïve iPSC (Fig. 3b,c). The results obtained from control and patient 2 EBs were not statistically significant but show similar tendency than patient 1 samples (Supplementary Fig. S3).

We also investigated the expression of *OCT4* pluripotency gene, *TFAP2C, D2-40, CD38*, and *KIT* early PGC markers genes as well as *DAZL* and *DDX4* late PGC markers genes by RT-qPCR; the results are presented in Supplementary Fig. S3.

**Figure 4:**
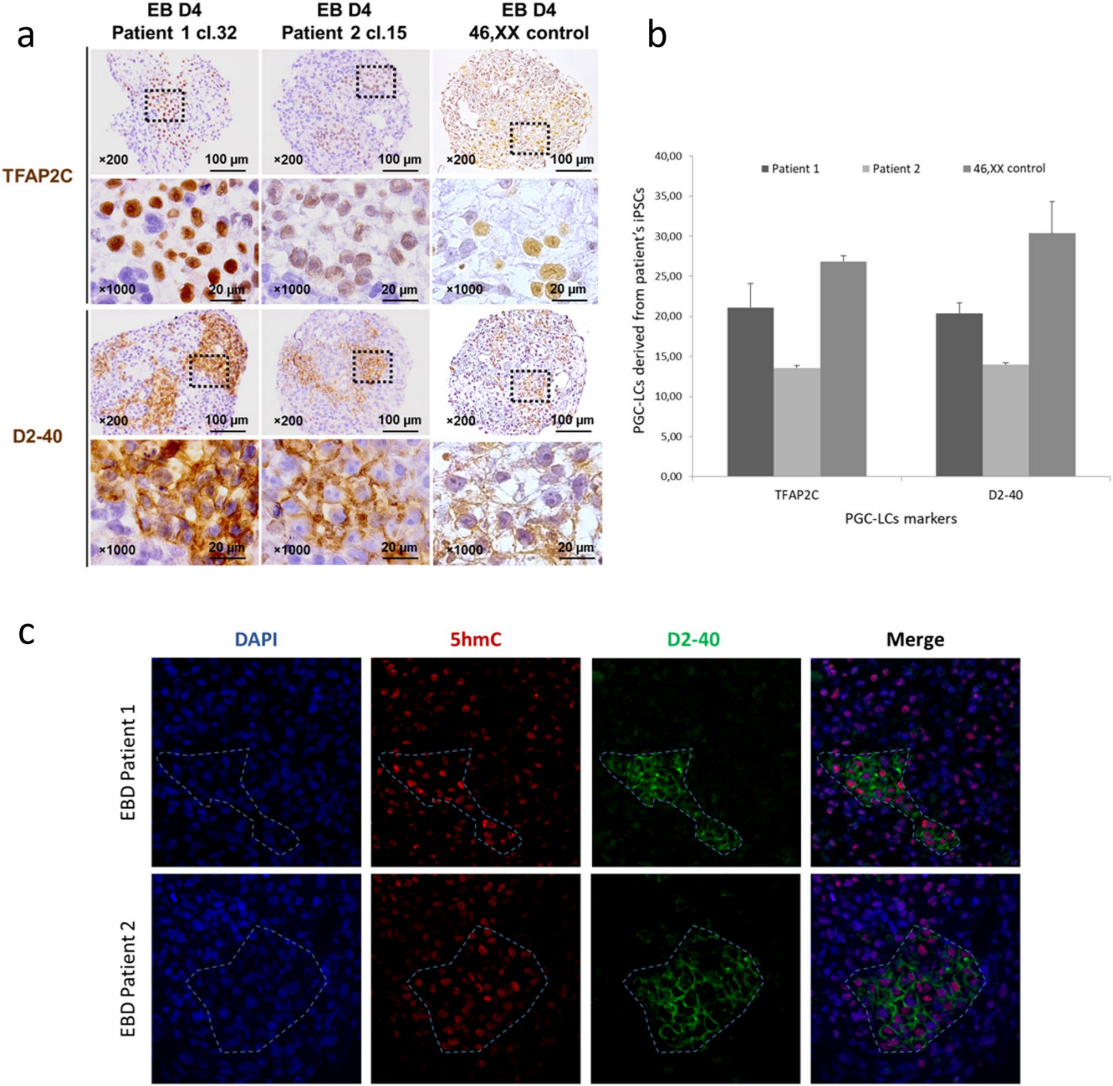
Immunohistochemical (IHC) expression of TFAP2C and D2-40 on EBs sections. The full protocol was repeated three times for the iPSCs of patient 1 and two times for the iPSCs of patient 2. (**a**) IHC pictures showing PGC-LCs positive for TFAP2C and D2-40 in EB D4 from patient 1 cl.32-hiPSCs and patient 2 cl.15-hiPSCs. (**b**) Percentage rate of PGC-LCs cells population differentiated within EBs from patient 1 cl.32-hiPSCs and patient 2 cl.15-hiPSCs as estimated after quantification. (**c**) Immunofluorescence (IF) showing localization of DAPI (blue), D2–40 (green) and 5-hydroxymethylcytosine (5hmC, red) in PGC-LCs and somatic cells in EBs at day 4. In order to clearly show the localization of PGC-LCs, we used the specific germ cell membranous marker D2-40 in combination with 5hmC for IF assay. Images show that, 5hmC is detectable in PGC-LCs in higher levels than in in somatic cells.

As *TFAP2C* and *D2-40* have been widely reported as key genes involved in human early germ cell development and represent suitable markers to identify hPGC-LCs, we performed immunohistochemical analysis with TFAP2C and D2-40 antibodies to assess the presence of PGC-LCs within the EBs^29–32^. TFAP2C^+^ and D2-40^+^ populations were estimated separately. For all clones tested, TFAP2C^+^ cells and D2-40^+^ cells were detected within EBs (Fig. 4a). TFAP2C^+^ positive cells in the EBs of patient 1 were estimated at 26.5 ± 3.5%, 6.8 ± 0.8%, and 30 ± 4.7%, while D2-40^+^ cells were estimated at 29.1 ± 2%, 5.3 ± 1%, and 27 ± 0.9%. In patient 2, TFAP2C^+^ cells were detected at 4.2 ± 1.8% and 23 ± 2.7%, while D2-40^+^ cells were estimated at 1.4 ± 1.2% and 26.5 ± 1.9%. Finally, in the control EBs (46,XX control), TFAP2C^+^ cells were detected at 26,8 ± 0.7%, while D2-40^+^ cells were estimated at 30,36 ± 3.7 (Fig. 4b). Therefore, we have demonstrated that iPSCs derived from infertile patients can efficiently differentiate into germ cell lineage, at least to the PGC-LC stage.

We next evaluated epigenetic reprogramming status of PGC-LCs by assessing proportion and distribution of DNA 5hmC in patient’s EBs cells by double immunostaining. Immunofluorescence assay show increased staining levels of 5hmC in PGCs compared to those of neighboring somatic D2-40^-^ cells in patient 1 and 2 EBs (Fig. 4c). Some surrounding D2-40^-^ cells show 5hmC staining, this could be explained by the fact that these cells are in a differentiation process towards germ cells lineage at day 4. The presence of 5hmC signals in PGC-LCs is consistent with the differentiation process toward germline lineage.

### Expression analysis of the candidate genes in patients 1 and 2

**Figure 5:**
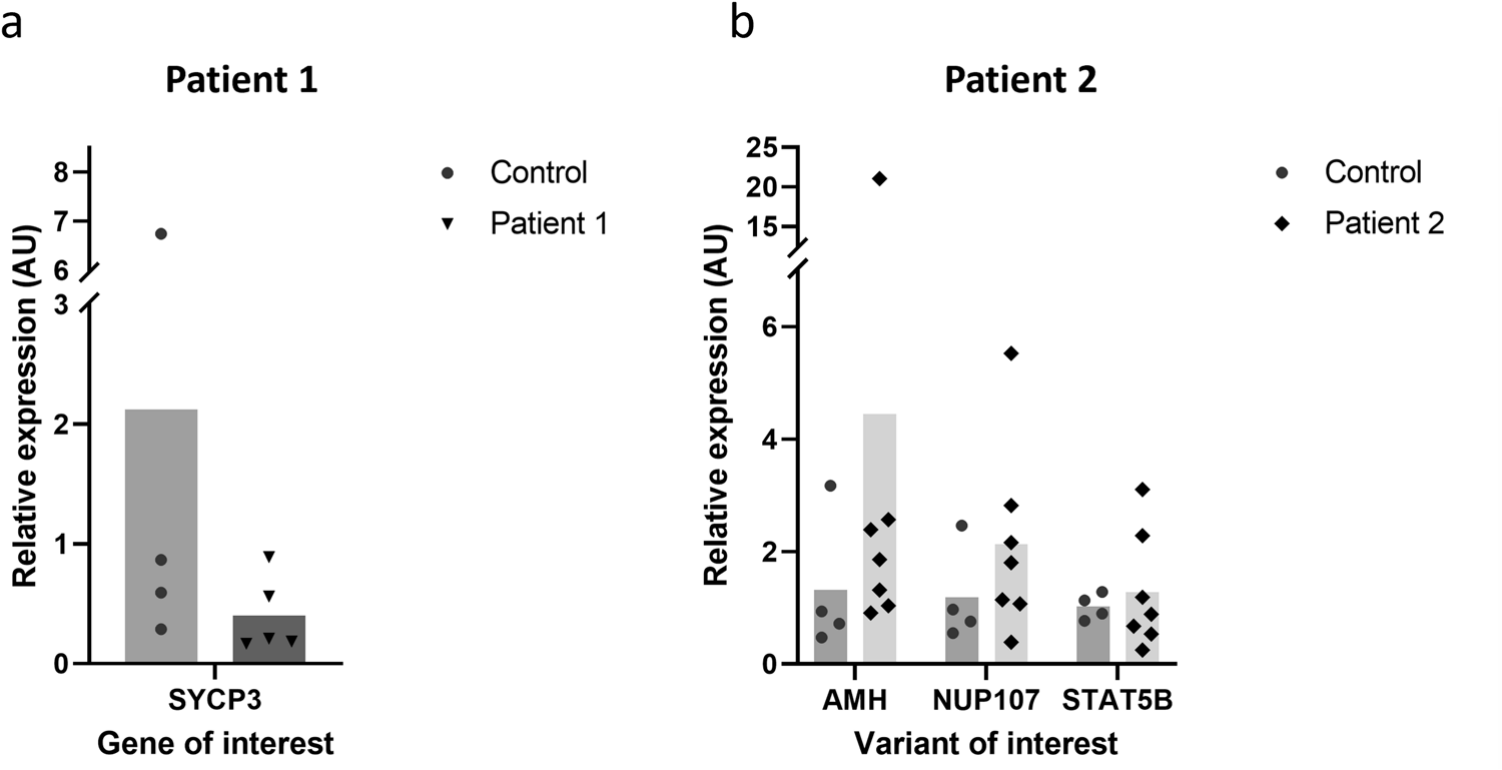
RT-qPCR analysis of candidate genes on embryoid bodies. (**a**) *SYCP3* expression level in patient 1 compared to 46,XX control cells. (**b**) *AMH, NUP107* and *STAT5B* expression levels in patient 2 compared to 46,XX control. Histograms represent mean from independent biological replicates represented by each point. Gene expression was normalized to RPLPO. Mann–Whitney tests were used for PCR analysis. *AMH* anti-Müllerian hormone, *NUP107* nucleoporin 107, *STAT5B* signal transducer and activator of transcription 5B, *SYCP3* synaptonemal complex protein 3.

In order to determine the potential involvement of the candidate genes identified by WGS in the phenotypes of patients 1 and 2, we evaluated their expression by RT-qPCR in the embryoid bodies of patient’s and control. Our results did not show a major alteration of *SYCP3* gene expression compared to control at least at PGC-LCs stage (Fig. 5a). We also investigated the expression status of the three candidate genes identified in patient 2 and found non-significant increase of *NUP107* and *STAT5B* compared to control. We observed a more marked upward trend in *AMH* gene expression in patient 2 EBs compared with the control (Fig. 5b). We compared AMH expression in primed iPSCs of patient 2 to that of two naive control iPSCs (46,XX and 46,XY); the results showed an intermediate level of *AMH* expression in patient 2 iPSCs compared to the *AMH* levels in 46,XX and 46,XY iPSCs controls (Supplementary Fig. S4).

## Discussion

To decipher the etiology of male infertility, we performed a full genomic characterization of genetic abnormalities of two cases of infertile men and identified potential loci and/or genes linked to infertility or sexual development. Through iPSC derived models, we indirectly showed that infertile men harboring complex genetic abnormalities could produce PGC-LCs. This result is novel and significant to the field, as it enables further investigation of the blockage and development process of gamete differentiation.

Patient 1 is a CCR carrier, which could alone explain the azoospermia, as the structural abnormalities might disrupt chromosome pairing at the pachytene stage during meiosis. Indeed, checkpoint mechanisms occur during the meiotic process. Failure of chromosome synapsis generally leads to spermatogenic arrest at the mid-pachytene stage of meiotic prophase or at metaphase stage of the first meiotic division, resulting in subfertility or infertility. Failure to detect such abnormalities in chromosome pairing can lead to aneuploidy, one of the major causes of embryonic loss. However, the testicular biopsy of patient 1 revealed the presence of few spermatocytes and extremely rare spermatids, attesting that the first and even the second division of meiosis occur despite the CCR, albeit at a low level. In patient 1, a breakpoint was identified at a distance of 300 kb from the *SYCP3* gene 3' end, but it did not occur in a known regulatory domain of the gene. Nevertheless, the breakpoint may have disrupted the regulatory elements of the gene and, therefore, its expression. However, our RT-qPCR results showed no major alteration of *SYCP3* expression in the patient 1 EBs**.** We cannot exclude that this is due to the fact that PGC-LC do not yet robustly express meiotic genes. This factor is a major player for germ cell differentiation as it enables the achievement of key processes, such as recombination and meiotic chromosomes segregation during gametes production; SYCP3 belongs to the synaptonemal complex that binds homologous chromosomes together at pachytene (synapsis), but was also found expressed in ESCs and iPSCs cells^33^. As heterozygous mutations in the *SYCP3* gene have been reported to be associated with azoospermia^34,35^, a mis-regulation of *SYCP3* expression in patient 1 could be involved in his spermatogenic failure. However, to determine whether the SNP identified in the *SYCP3* gene had an impact on the patient's 1 infertility, further experiments are required including differentiation towards meiotic or more mature germ cells and forced expression of *SYCP3* using specific lentiviral vector for instance.

The clinical picture of the 46,XX DSD patient and level of testosterone are consistent with testicular tissue development: either testis development or the co-existence of both testicular and ovarian tissue in the same gonad (ovotestis). In any event, the patient’s testicular tissue produced, in utero, insufficient testosterone for the full masculinization of the genitalia, leading to the ambiguous external genitalia and impaired development of Wolffian duct derivatives.

WGS, as well as the array-CGH, enabled confirmation of the 46,XX karyotype without *SRY* sequences in the entire genome of the patient. Although the WGS approach succeeded in identifying several genes potentially involved in the patient's phenotypic traits, the causative gene or the molecular mechanisms responsible for the patient’s sexual reversion were not highlighted. While some DSD cases can be explained by a single causal variant*,* many other cases remain unexplained, potentially resulting from different mutations, which, in combination, have tilted the balance towards male differentiation.

Three rare variants of potential interest that could be linked to the patient’s phenotype were identified by WGS, namely *STAT5B*, *NUP107* and *AMH* genes. However, no significant change in *STAT5B* gene expression was observed in patient’s EBs compared to the control EBs. *STAT5B* is involved in many biological processes such as severe growth hormone insensitivity, resulting in short stature (OMIM#245590)^36^. Patient 2 harbors a rare missense mutation within exon 7 of the *STAT5B* gene. The patient’s height was 1.55 m, suggesting that the *STAT5B* mutation might be involved in this specific phenotype.

The expression of AMH and to a lesser extent NUP107 is slightly increased in DSD EBs compared to control EBs 46,XX, but without statistical significance**.** The *NUP107* variant (exon 12) is present at a low frequency in the gnomAD database (2.122 10^–5^) and represents a potential variant of interest. A rare homozygous missense variant in exon 12 of the *NUP107* gene (c.1063C>T, p.R355C) has been reported in two sisters affected with primary amenorrhea and hypergonadotropic hypogonadism^37^. However, the results do not support a disruption of *NUP107* gene expression at least at PGCs level.

AMH plays a critical role in the normal sexual differentiation and can be useful in the initial evaluation of DSD suspicion in childhood. But AMH expression in the diagnosis of DSD at adult age has not been to date investigated. However, we know that in male, the onset of puberty lead to the downregulation of AMH expression due to the increased intratesticular production of androgens and its receptor. After puberty, in female, the level of expression of AMH remain relatively stable until the third decade of life. We assessed the expression of *AMH* gene in primed iPSCs from patient 2 and two control iPSCS (46,XX and 46,XY). The level of *AMH* expression in the DSD cells is intermediate between male and female control iPSCs, suggesting that *AMH* expression could have contributed to the abnormal Müllerian duct development in this 46,XX patient in whom female internal reproductive organs were reduced to a vaginal cavity segment. Nevertheless, the *AMH* variant alone is not sufficient to explain sexual reversion. AMH is expressed by Sertoli cells and is involved in the regression of the Müllerian duct, which would otherwise develop into uterus, fallopian tubes and upper vagina. Thus, in the context of our patient DSD 46,XX, the abnormal increase in *AMH* expression could explain the presence of residual Müllerian tissue and, more importantly, is consistent with the presence of testicular tissue in his gonad.

Specific phenotypes, such as DSD syndrome, are more likely the result of multiple genetic factors, however those leading to atypical sex development are often not identified due to insufficient knowledge of the pathogenesis and underlying mechanisms. Thus, it is conceivable to consider that additional mutations have contributed to the DSD phenotype of patient 1.

To model reproductive pathologies from these complex genetic landscapes we first assessed to differentiate PGC-LCs from primed iPSCs, but all our attempts failed (data not shown). Since the naive pluripotent state overcomes several hurdles encountered by primed pluripotent state, such as differentiation capability, single‐cell passaging, and low gene editing efficiency^38^, we derived, for the first time in our knowledge, naive iPSCs from these two patients. So far, two strategies based on the forced expression of specific genes and/or the addition of specific factors in the medium, were used to induce male germ cells in vitro. Previous studies have demonstrated the capacity of normal hiPSCs to differentiate into PGCs^17,19,20^, and into gonocytes^22^, SSCs^39^, and meiotic germ cells^18,19,39–41^. To date, PGC-LCs or mature germ cells within the context of karyotype abnormalities have not been generated. In our study, naive-iPSCs were successfully differentiated into PGC-LCs through EB formation in the presence of the essential BMP2/4, LIF, EGF, and SCF factors. Indeed, we revealed the high expression of TFAP2C and D2-40 early PGC markers. Remarkably, *SOX17*, the human-specific key regulator of PGC-LC specification, was shown to be significantly upregulated within EBs^21,42,43^, indicating that our culture conditions were effective in inducing hPGC-LCs from both patient’s naive iPSCs.

Even if the proportion of positive germ cells within EBs were variable, the PGC fate was achieved by all differentiation assays. Furthermore, hPGC-LCs of up to 30% were generated, which are comparable to the study by Irie and colleagues who obtained approximatively 46% of hPGC-LCs in a normal genetic context^21^. We could also show an increase of DNA 5hmC staining in the PGC-LCs through germ cells differentiation process from infertile patient’s iPSCs, confirming the successful derivation of PGC-LCs from those patients. These results indicate that the complete loss of fertility of the two patients may not be attributed to PGC formation abnormalities. Furthermore, human PGC-LCs were demonstrated to be produced from both XX and XY cells in male infertility with meiotic arrest and gonadal sex reversal.

Regarding patient 1, since *SYCP3* gene is known to be highly expressed during meiosis, further differentiation of iPSCs-derived PGCs through meiosis is necessary to determine whether there is an abnormal *SYCP3* expression beyond the PGC stage, and, therefore, its potential involvement in altered meiosis. Driving germinal differentiation further may provide an answer for the underlying cause of meiotic arrest and a better understanding of CCR pairing and checkpoint mechanism behavior during meiosis. In mice, functional gametes leading to healthy and fertile mice can be generated from iPSC derived PGC-LCs. However, the use of human PGC-LCs for xenografts in animal models or fecundation and progeny development to investigate whether these cells could give rise to functional gametes is not realistically feasible due to legal, philosophical, societal, and ethically evident challenges, even in the context of infertility.

For patient 2, we assume that the *SRY*-negative 46,XX PGC-LCs generated from his own iPSCs would most likely be unable to undergo meiosis, as the Y chromosome plays a major role in male fertility. Indeed, deletions of specific regions of the Y chromosome were linked to early failure of spermatogenesis and, consequently, to infertility^44–46^. For example, microdeletions of the AZF regions in Yq11 represent one of the most frequent genetic causes of azoospermia or severe oligospermia^47^. Therefore, it remains to be evaluated whether the PGC-LCs of patient 2 could undergo meiosis to differentiate into more mature male germ cells if AZF sequences are added. In view of this, an interesting question is raised, as it may be more conceivable to produce oocytes from the XX male’s PGCs. This alternative would raise evident ethical concerns and question the acceptability of such an alternative for the patient. Hence, this highlights the absolute need for reflection and debate generated by such a new technology. Such an option also requires better knowledge of gene expression and epigenetic modifications in germ cells, which have been previously poorly studied because of the lack of an appropriate model, and can now be investigated as a result of the generation of PGCs from infertile men in the present study.

In conclusion, our results indicate that naive iPSCs derived from two infertile patients characterized by important genetic abnormalities are capable of efficient production of PGC-LCs. However, it remains to be elucidated whether *SYCP3* (patient 1), *AMH* and *NUP107* (patient 2) are involved in the absence of mature gametes in the patients. Nevertheless, these results are extremely encouraging and reinforce the need to further decipher the molecular mechanisms responsible for the lack of gametes. Moreover, we believe that the naive iPSC model introduced in this study would be useful to explore new therapeutic strategies, such as high-throughput screening of drug molecules, to overcome the blockage of gamete differentiation.

## Materials and methods

### Patients and controls

The patient 1 was 38 years old and consulted for infertility after he and his partner had been trying to conceive for 2 years. The patient was the first child of unrelated parents, and he had four brothers and five sisters whose fertility status could not be determined because of their personal situations (they were younger and not actively trying to procreate). Clinical examination excluded obstruction of the genital tract but revealed marked bilateral atrophy of the gonads, with a testicular volume of only 7 cm^3^ (normal range: 20–25 cm^3^). Semen was collected after a requested five-day period of abstinence. Two spermograms performed eight months apart revealed azoospermia. Testicular doppler ultrasound and ultrasound scans of the deep genital tract showed hypervascularization of the prostate, with calcification of the central zone, slight differential thickening of the pelvic walls, but without obstruction, and non-retentive vesicles. Laboratory tests revealed hormonal dysregulation, with a low serum concentration of inhibin B (36 pg/mL; normal range: 80–270 pg/mL) and a high serum concentration of FSH (15.5 IU/L; normal range: 1.4–10 IU/L). The serum concentration of LH was normal (7.9 IU/L; normal range: 1.4–8 IU/L). Bilateral testicular biopsy was performed, and histological analysis showed maturation arrest in all the seminiferous tubules mostly at the spermatocyte stage and more rarely at the spermatid stage. Thus, meiosis was initiated but not completed. In addition, testicular Leydig’s cell hyperplasia was observed.

The patient 2 was 44 years old and belongs to a consanguineous family as his parents were first cousins. Compared to other members of his family the patient has a smaller size. At birth, he presented an uro-genital malformation associated with persistence of a large vaginal cavity (4–5 cm deep) implanted in the posterior urethra below the sphincter, requiring many chirurgical interventions. At the age of 21, the examination showed a small size with a male-oriented phenotype, a small rod (6 cm long), two atrophic gonads (left and right gonad with a volume of respectively 2 ml and 0.9 ml) with a scrotal position about 1 cm high. Radiological and endoscopic explorations were carried out, revealing the persistence of the vaginal cavity of uro-genital sinus type, implanted in the posterior urethra below the sphincter. Hormonal examination was performed, revealing high FSH (33UI/L; normal range: 1.4–10 IU/L) and LH (56 UI/l; normal range: 1.4–8 IU/L) values, and low serum testosterone concentrations (1.4 ng/ml; normal range: 2.50–9.50 ng/dL). The implantation of the urogenital sinus on the posterior side of the urethra has been shown to cause episodes of urinary incontinence, recurrent urinary infections, dysuria and burning urination by infections. In addition, the patient had sexual problem with retrograde ejaculation. The ablation of this müllerian residue was therefore carried out at the age of 33 years old. Unfortunately, despite our request, the parents were not available.

A written informed consent was obtained from each patient for the use of its clinical data and biological samples for genetic research and publication purposes. They were specifically informed that the genetic research would concern constitutional and pathological genetics. All methods were performed in accordance with French Ethics law, and case reports do not need to go through Ethics committees according to French Law. The experimental protocol was approved by the Assistance Publique-Hôpitaux de Paris institutional committee.

PB12.CO3 (iPSCs 46,XX control, kindly provided by C. Monville) and UHOMi002-A (iPSCs 46,XY control, PUBMED: 33099111) were derived from two fertile individuals and were used for RT-PCR analysis. 46,XX iPSCs control line were used for the derivation toward naive state of pluripotency and for the differentiation of PGC-LCs following the same experimental protocol used for patient 1 and 2. We were not authorized to use 46,XY iPSCs line for PGC-LC differentiation, but we could use them for RT-qPCR analysis at primed and naive stage.

### Conventional and molecular cytogenetic analysis

Standard chromosomal analyses were performed on cultured peripheral lymphocytes from both patients and on derived iPSC clones, by standard procedures [G-banding with trypsin using Giemsa (GTG); R-banding after heat denaturation and Giemsa (RHG)].

FISH analyses were performed on metaphase spreads of lymphocytes from the patient 2. The following probes were used, in accordance with the manufacturer’s recommendations: centromeric probes specific for chromosomes 18, X and Y (Vysis) and *SRY* probes [The probe mix also contains control probes for the X centromere (DXZ1), and for chromosome Y (DYZ1, the heterochromatic block at Yq12)] (Cytocell). Images were created with the CytoVision 7.0 Software, Leica Biosystems.

Genomic DNA was isolated from both patient’s peripheral blood and from derived iPSCs, with the Maxwell® 16 Blood DNA Purification Kit (Promega, Biotech). The concentration and quality of the extracted DNA were evaluated with a NanoDrop ND-1000 spectrophotometer (NanoDrop Technologies).

Genomic imbalances from patient 1 and 2 were analyzed by oligonucleotide based array-comparative genomic hybridization analysis (array-CGH). A hight resolution 1 M microarray and a 400 K custom microarray were used for each patient (Agilent Technologies, Massy, France). The 1 M microarray covered whole genome with an intragenic resolution of 1.8 kb. The custom design had a whole genome coverage with a 5 kb resolution and was strongly enriched in 363 genes with an exonic resolution of 0.1 kb and an intronic resolution of 0.6 kb. These genes of interest were selected because of their certain or potential involvement in reproductive function and sexual development in both humans and mice. Hybridization was performed according to the manufacturer’s protocol. Images processing and data analysis were performed with CytoGenomics software (4.0.3.12) (Agilent Technologies). The ADM2 algorithm was used for statistical analysis. Copy number variations were considered significant if they were defined by tree or more oligonucleotides and were not identified in the Database of Genomic Variants as a polymorphism (http://projects.tcag.ca/cgi-bin/variation/gbrowse/hg19).

### Short-read whole genome sequencing (patient 1)

Genome sequencing (GS) was performed with 150-bp paired-end reads using the Illumina HiseqX Ten platform. Reads were aligned to the human genome reference (hg19) using the Burrows-Wheeler aligner. Base quality scores were recalibrated using GATK v3.8. Candidate breakpoints were predicted using Lumpy for inversions and translocations and Control-FREEC for dup/del^48^. Variants were filtered based on population frequency and their presence in public databases. Candidate breakpoints were visually inspected and selected using the Integrative Genomic Viewer (IGV) tool.

GS successfully yielded 40 × mean read depth across the whole genome. SVs were analyzed using “in house” pipeline, a local pipeline combining Lumpy and Control-FREEC. The outputs of the callers, a tabulation separated file, was annotated using public databases such as DGV, GnomAD SV, Developmental Delay, and ISCA Using GS data, Lumpy predicted 1705 structural variations breakpoints, including 958 chromosomal translocations, 276 inversion and Control-FREEC predicted 471 deletions and duplications.

### Whole genome sequencing (patient 2)

WGS was performed for patient with DSD by the Centre National de Recherche en Génomique Humaine (Institut de biologie François Jacob, CEA). After a complete quality control, genomic DNA (1.1 µg) has been used to prepare a library for whole genome sequencing, using the Illumina TruSeq DNA PCR-Free Library Preparation Kit, according to the manufacturer's instructions. After normalization and quality control, qualified libraries have been sequenced on a HiSeqX5 platform from Illumina (Illumina Inc., CA, USA), as paired-end 150 bp reads. 1 lane of HiSeqX5 flow cell has been produced for each sample, in order to reach an average sequencing depth of 30 × for each sample. Sequence quality parameters have been assessed throughout the sequencing run and standard bioinformatics analysis of sequencing data was based on the Illumina pipeline to generate FASTQ file for each sample.

After demultiplexing, sequences were aligned to the reference human genome hg19 using the Burrows-Wheeler Aligner^49^. Downstream processing was carried out with the Genome Analysis Toolkit (GATK), SAMtools, and Picard, following documented best practices (http://www.broadinstitute.org/gatk/guide/topic?name=best-practices). Variant calls were made with the GATK Haplotypecallers version 3. Structural variants were assessed using Manta^50^, Canvas^51^ and WisecondorX^52^. The annotation process was based on the latest release of the Gencode database (v31)^53^, Gnomad (v2.1)^54^, Clinvar (v20190815), CADD (v1.4)^55^. Variants were annotated and filtered using the Polyweb software interface designed by the Bioinformatics platform of University Paris Descartes.

### Patients-derived iPSCs generation and pluripotency characterization

Patient 2 specific iPSCs were generated by reprogramming his erythroblasts using Sendai virus-mediated gene transfer and specific iPSCs derivation was realized as previously described^24^. PCR and immunofluorescence analysis were assayed to assess endogenous expression of pluripotency marker genes in patient 2 specific iPSCs. In vivo assessment of pluripotency was performed by teratoma formation and histological analysis. Animal experiments were performed according to protocols approved by the local animal ethics advisory committee, registered at the French research ministry in accordance with French national regulation (national transposition of European directive 2010/63/CE). Mice experimentation was approved by the Commissariat à l'Energie Atomique et aux Energies Alternatives, 92265 at Fontenay aux Roses, France.

### In vitro derivation of naive hiPSCs from conventional hiPSCs

Primed-hiPSCs of both patients were maintained on irradiated mouse embryonic fibroblasts (MEFs) in DMEM/F12 GlutaMAX supplemented with 20% KSR (thermofisher), 1X non-essential amino acids, 50 µM β-mercaptoethanol (thermofisher), and 10 ng/ml of bFGF (thermofisher). Media were replaced every day. Cells were passaged as clumps every 4 to 6 days using trypsin and 10 µM of ROCK inhibitor (TEBU-Bio). Primed-like hiPSCs were converted into a naive state by cultivating them on MEFs feeders cells in knock-out DMEM medium (thermofisher) supplemented with 20% KSR (thermofisher), 2 mM L-Glutamine (thermofisher), 0.1 mM MEM NEAA (thermofisher) and 50 µM β-mercaptoethanol (thermofisher) and containing basic fibroblast growth factor (b-FGF, thermofisher), human leukemia inhibitory factor (LIF, Peprotech), transforming growth factor-β1 (TGFβ, TEBU-Bio), and four small inhibitory molecules (4i), 3 μM CHIR99021 (R&D systems), 1 μM PD0325901 (R&D systems), 5 μM SB203580 (R&D systems), and 5 μM SP600125 (R&D systems)^27^. Cells were maintained in 4i hiPSC medium for two weeks replacing daily with fresh medium. The growing naive-hiPSCs colonies were picked up and passaged every 2–3 days as single cells with accutase. For each passage, 10 μM of ROCK inhibitor (TEBU-Bio) were used.

### In vitro induction of PGC-LC from hiPSCs

Naive-hiPSCs colonies were separated as single cells with accutase and seeded in ultra-low attachment U-bottom 96 well plate (Corning, New York, USA) at a density of 8000–10,000 cells/well in PGC-LCs medium. Then naive-hiPSCs were centrifuged 120 g for 2 min to induce embryoid bodies (EBs) formation. Simultaneously of EBs formation, naive-hiPSCs differentiation into PGC-LCs was induced by culturing EBs in GMEM medium (thermofisher) supplemented with 15% KSR (thermofisher), 2 mM L-Glutamine (thermofisher), 0,1 mM nonessential amino acid (thermofisher), 1 mM sodium pyruvate (Sigma), 50 µM mercaptoethanol (thermofisher), 100 U/ml Penicillin—100 U/ml Streptomycin (Sigma) and cytokines 500 ng/ml BMP4 (TEBU-Bio), 1 µg/ml LIF (Peprotech), 100 ng/ml SCF (TEBU-Bio), 50 ng/ml EGF (TEBU-Bio) and 10 µM ROCK inhibitor (TEBU-Bio). After 4 days, EBs were observed in the center of each well. Three to five EBs were formaldehyde-fixed and paraffin-embedded for each clone studied, then paraffin sections of EBs were used for immunohistochemistry experiments.

### Immunohistochemical staining for TFAP2C and D2-40

Four to five sections from each EBs clone were mounted on slides. The paraffin was removed, and sections were rehydrated in several baths of toluene, ethanol 100°, ethanol 96°, H_2_O and finally stained with hematoxylin–eosin. Endogenous peroxidase activity and non-specific protein binding were blocked by incubating the sections in H_2_O_2_ followed by normal horse serum 2.5%. The sections were then incubated with TFAP2C or D2-40 mouse primary antibodies for 1 h at 37 °C. Primary antibody was detected by incubation with anti-mouse secondary antibody. Finally, peroxidase activity was detected with DAB. Sections were rinsed in PBS between each step. Analyses and quantification were performed with Histolab version 11.5.1 Software (Microvision Instruments, https://www.microvision.fr) analysis software.

### Immunofluorescent staining and imaging analysis

For immunofluorescence staining (5hmC, D2-40), paraffin sections of EBs were submitted to antigen retrieval with tris–EDTA (121 °C, 20 min) and then blocked in 0,2% gelatine, 0.05% Tween 20, 0.2% BSA and PBS 1X for one hour before adding antibodies. The primary antibodies used in this study were as follows: monoclonal rabbit anti-5hmC (Active Motif ref #39769; 1:400) and monoclonal mouse anti-D2-40 (Dako, ref M3619, 1:200). Specific rabbit and mouse secondary antibodies were conjugated respectively with Alexa Fluor 594 and 488 (1:500). Slides were mounted in Vectashield medium. Image acquisition was accomplished with a laser-scanning confocal microscope (confocal spinning disk, W1)) and images were analysed using MetaMorph device (Molecular Devices, https://fr.moleculardevices.com) and ImageJ 1.52 (National Institutes of Health, https://imagej.nih.gov) software.

### RNA extraction and reverse transcription reaction

Total RNA was extracted using RNeasy mini kit (Qiagen, Valencia, CA, USA) according to the manufacturer's instructions. Reverse transcription was carried out using High capacity kit (Applied biosciences, Foster City, CA, USA) following the instructions provided by the manufacturer. Maximal amount of 1 µg of RNA was used in RT reactions. cDNA synthesis in 20 µL of total volume was as follows: 2 µl of 10 × RT random primers, 2 µl of 10 × RT buffer, 1 µl of RNAse inhibitor, 0.8 µl of 25 × dNTP Mix (4 mM) and 1µL of 50 U/µl MultiScribe Reverse Transcriptase completed by nuclease free H2O up to 20 µl. After gently mixing tube contents and incubating at 25 °C for 10 min, the cDNA synthesis was performed at 37 °C for 2 h followed by 85 °C for 5 min to inactivate the reverse-transcriptase. Diluted or undiluted cDNA was used in qPCR immediately, or stored at − 20 °C until use.

### Real-time PCR

StepOne Plus Real-Time PCR System (Applied Biosystems, Foster City, CA, USA) and SYBR-green labelling were used for quantitative RT-PCR. Three µl of cDNA (1:10 diluted) were used for duplicate amplification in a 10 µl reaction solution with 0.5 µM (5 pmol) of each primer. The following PCR program was used: 95 °C for 5 min, 40 amplification cycles at 95 °C for 10 s, 60 °C for 30 s. Serial dilutions of each studied transcript were used to determine the amplification efficiency of each target and housekeeping gene (RPLP0). In the present study, data are obtained by analysis with StepOne Software 2.3 and are presented as the fold-change in target gene expression normalized to the internal control gene *RPLPO*. Error bars indicate mean ± SD from two to five independent biological replicates. The average threshold cycle (CT) was calculated for both the target gene and *RPLP0*, and ∆CT was determined as [the mean of the duplicate CT values for the target gene]-[the mean of the duplicate CT values for RPLPO]. ∆∆CT represented the difference between the paired samples, as calculated by the formula ∆∆CT = (∆CT of sample − ∆CT of control). For induction of PGCLCs from various iPS lines, mRNA expression was normalized to the mean expression from all naive iPSCs in order to compare expression both among the various iPSCs and among the EBs. Means were performed using GraphPad Prism version 9.2.0 for Windows, GraphPad Software, San Diego, California USA (www.graphpad.com). Data represent the fold change between gene expression in each sample as compared to the mean expression among the naïve IPSCs.

RT-qPCR was performed with primers specific for *OCT3/4* (sense primer 5′-CTGCAGCAGATCAGCCACAT-3′ and reverse primer 5′-CACATCCTTCTCGAGCCCAA-3′), *NANOG* (sense primer 5′-TGGCCGAAGAATAGCAATGG-3′ and reverse primer 5′-AGTCGGGTTCACCAGGCAT-3′)*, SOX17* (sense primer 5′-AAGGGCGAGTCCCGTATC-3′ and reverse primer 5′-CTTGTAGTTGGGGTGGTCCT-3′)*, NANOS3* (sense primer 5′-AGGAGCAGGTTTCAGAGGTGC-3′ and reverse primer 5′-AGAGCAGGAGGGCGAAGG-3′)*, CD-38* (sense primer 5′-GGCGCGATGCGTCAAGTACACTG-3′ and reverse primer 5′-CCTAGCAGCGTGTCCTCCAGGGTG-3′)*, TFAP2C* (sense primer 5′-GTACGAAGAGGACTGCGAGGA-3′ and reverse primer 5′-GGATTCCCATTGCTGCTCC-3′)*, c-KIT* (sense primer 5′-TGAATCTACTTGGAGCCTGCAC-3′ and reverse primer 5′-CTGTAATGACCAGGGTGGGC-3′)*, D2-40* (sense primer 5′-TGTGGTTATGCGFAAAAATGTCG-3′ and reverse primer 5′-CCTTCAGCTCTTTAGGGCGAG-3′)*, DAZL* (sense primer 5′-TACTCCACCCTCTGGAAATGG-3′ and reverse primer 5′-GCTTCGGTCCACAGATTTCTT-3′)*, REX1* (sense primer 5′-CAGTCCAGCAGGTGTTTGC-3′ and reverse primer 5′-GCATTCTATGTAACAGTCTGAGA-3′)*, DDX4* (sense primer 5′-GCCTCTGGGCGGAATTTT-3′ and reverse primer 5′-CGCTTATTACACTCACCAGCATG-3′), *AMH* (sense primer 5′- CATCCCCGAGACCTAC-3′ and reverse primer 5′- GGACCTGCATCTTCAG-3′), *NUP107* (sense primer 5′- GTTTGTTGATGGAGGGTG-3′ and reverse primer 5′- CTCAGAGGATACCATATCTG-3′), *STAT5B* (sense primer 5′- CTACGTGTTTCCTGATCG-3′ and reverse primer 5′-CCACTTGCTTGATCTGTG-3′), *SYCP3* (sense primer 5′- GAGCCTATGACTTTGAGACTG-3′ and reverse primer 5′-CAACTACTCCTGCAGAAGACC-3′), *KLF4* (sense primer 5′- GAGTTCCCATCTCAAGGCAC-3′ and reverse primer 5′- GCGAATTTCCATCCACAGCC-3′), *TFCP2L1* (sense primer 5′-GACAGCAACCTGTCTGTGTAC-3′ and reverse primer 5′- GTTGGCGATCTTCTCAATCAGC-3′) and *RPLPO* (sense primer 5ʹ-TCCCACTTGCTGAAAAGGTCA-3ʹ and reverse primer 5ʹ-CAAAGGCAGATGGATCAGCC-3ʹ).

## Supplementary Information

Supplementary Figure 1.

Supplementary Figure 2.

Supplementary Figure 3.

Supplementary Figure 4.

Supplementary Figure 5.

Supplementary Legends.
