# Supplementary figures and images for: iPSCs derived from infertile men carrying complex genetic abnormalities can generate primordial germ-like cells
Source: Sci Rep. 2022 Aug 22;12:14302. doi: 10.1038/s41598-022-17337-2 (PMC9395518; doi:10.1038/s41598-022-17337-2)

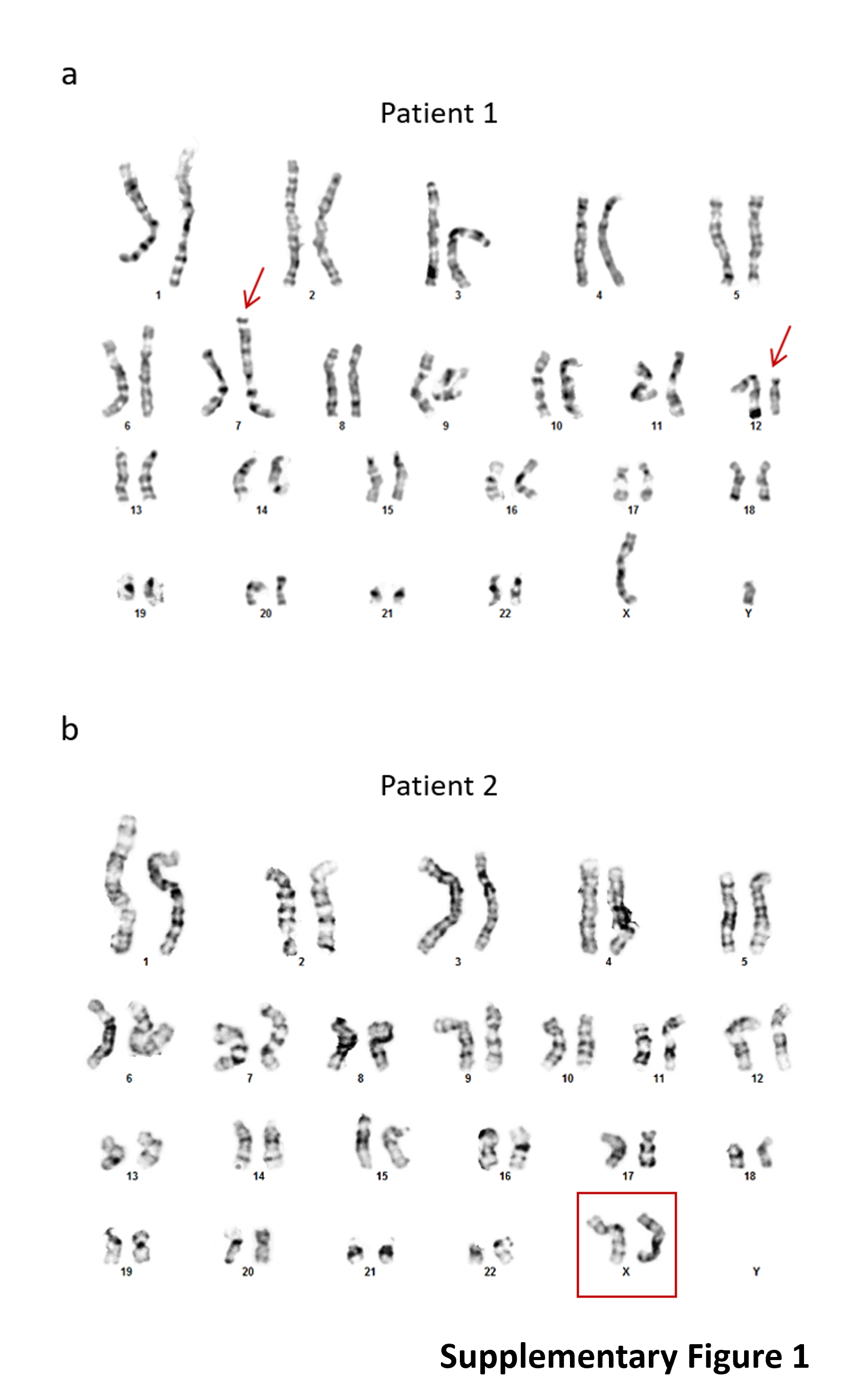

Supplement: Supplementary file 1 — Supplementary Figure 1. [file 41598_2022_17337_MOESM1_ESM.tif]

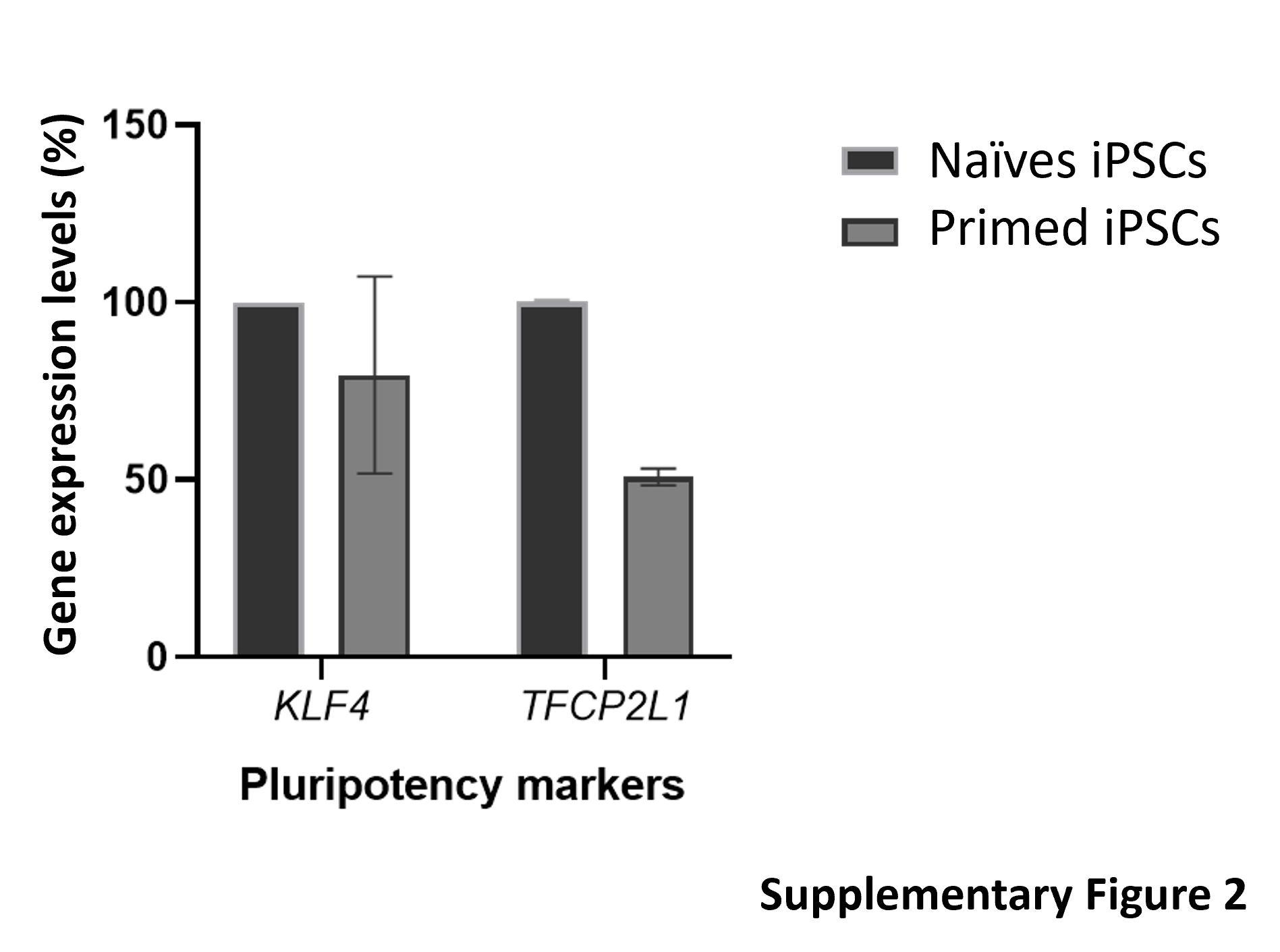

Supplement: Supplementary file 2 — Supplementary Figure 2. [file 41598_2022_17337_MOESM2_ESM.tif]

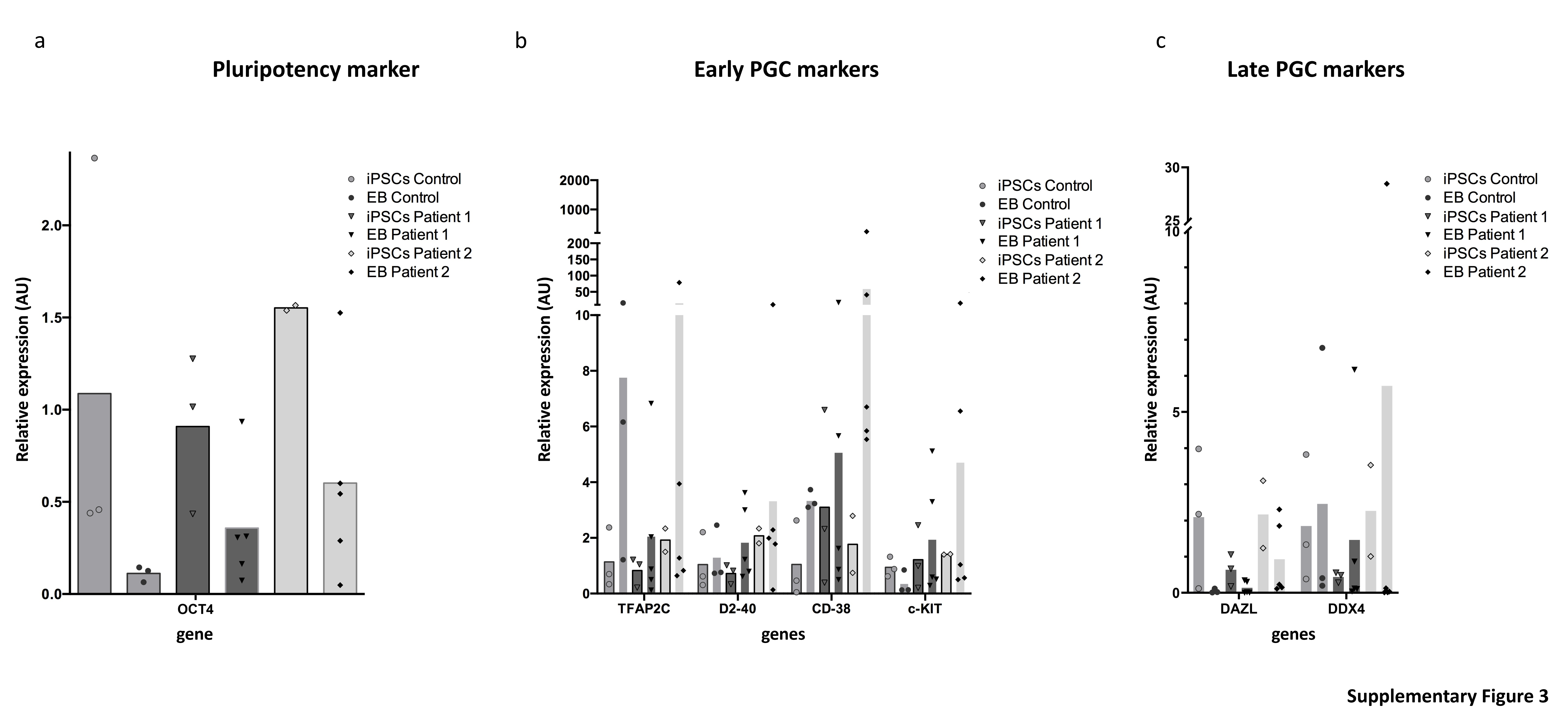

Supplement: Supplementary file 3 — Supplementary Figure 3. [file 41598_2022_17337_MOESM3_ESM.tif]

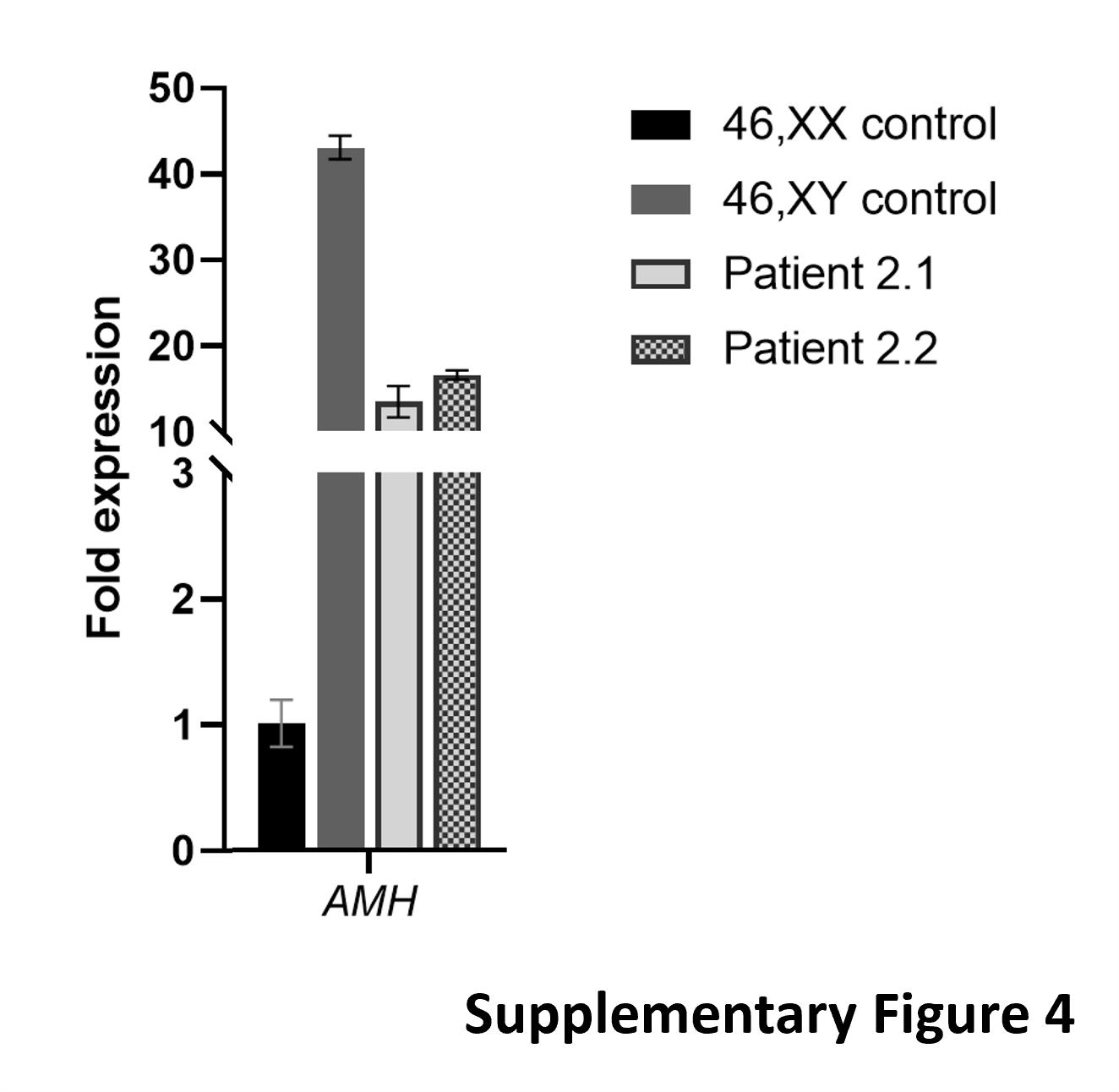

Supplement: Supplementary file 4 — Supplementary Figure 4. [file 41598_2022_17337_MOESM4_ESM.tif]

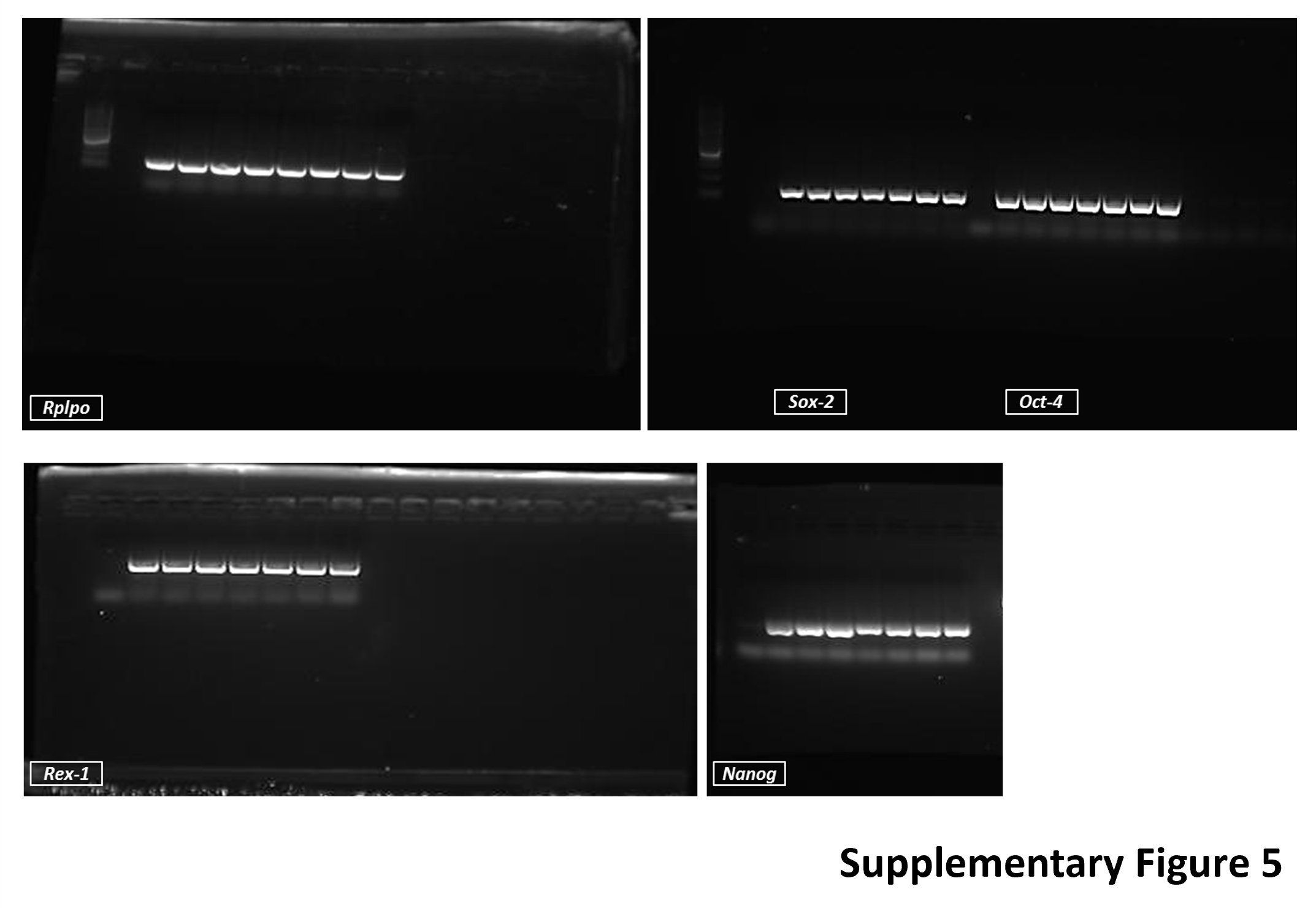

Supplement: Supplementary file 5 — Supplementary Figure 5. [file 41598_2022_17337_MOESM5_ESM.tif]
