# Supplementary material for: iPSCs derived from infertile men carrying complex genetic abnormalities can generate primordial germ-like cells
Source: Sci Rep. 2022 Aug 22;12:14302. doi: 10.1038/s41598-022-17337-2 (PMC9395518; doi:10.1038/s41598-022-17337-2)
Supplement: Supplementary file 6 — Supplementary Legends. [file 41598_2022_17337_MOESM6_ESM.docx]

**Supplementary information**

# Supplementary Fig. 1. Chromosomal stability investigation of iPSCs clones from patient 1 and 2 through reprogrammation and culture. (a) Patient 1 karyotype showing complex structural rearrangment of chromosomes 7 and 12 without additionnal chromosomal abnormatities. Red arrows indicate the rearrangements. (b) Patient 2 karyotype revealing a 46,XX female karyotype without additionnal chromosomal abnormatities. Red square show the two X chromosomes. Images were created with the CytoVision 7.0 Software, Leica Biosystems (https://www.leicabiosystems.com).

**Supplementary Fig. 2. Evaluation of primed to naïve conversion by quantitative RT-PCR analysis of Klf4 and TFCP2L1 markers.** TFCP2L1 and to a lesser extent KLF4, show increased expression in the converted patient’s naive iPSCs compared to primed patient’s iPSCs. Data were analysed using StepOne Software v2.3 (Life Technologies, https://www.thermofisher.com). Figures, means and standard deviations were obtained using GraphPad Prism version 9.2.0 for Windows, GraphPad Software, San Diego, California USA ([www.graphpad.com](http://www.graphpad.com)).

**Supplementary Fig. 3. EB differentiation and gene expression analysis.** RT-qPCR analysis for detection of the pluripotency marker gene *OCT4* (**a**); early PGC marker genes *TFAP2C, D2-40, CD-38* and *c-KIT* (**b**); late PGC marker genes (**c**)*.* Relative expression levels are shown with normalization to *RPLPO* gene. Histograms represent mean from independent biological replicates represented by each point. Mann-Whitney tests were used for PCR analysis.

Abbreviations: EB: Embryoid Body; iPSCs: Induced Pluripotent Stem cells.

**Supplementary Fig. 4. AMH candidate gene analysis using genomic quantitative RT-qPCR**. AMH normalized expression levels in patient 2 primed iPSCS compared to 46,XX and 46,XY primed iPSCs controls. AMH: Anti-Müllerian Hormone. Data were analysed using StepOne Software v2.3 (Life Technologies, https://www.thermofisher.com). Figures, means and standard deviations were obtained using GraphPad Prism version 9.2.0 for Windows, GraphPad Software, San Diego, California USA ([www.graphpad.com](http://www.graphpad.com)).

# Supplementary Fig. 5. Full length gels presented in Fig. 2a.
